# MMP-3 and TIMP-1 as prognostic biomarkers in VZV-induced retinal necrosis

**DOI:** 10.3389/fcimb.2024.1502912

**Published:** 2024-12-12

**Authors:** Zhujian Wang, Yu Liu, Min Zhou, Boya Lei, Qing Chang, Wenjun Cao

**Affiliations:** ^1^ Department of Laboratory Medicine, Fudan University Eye Ear Nose and Throat Hospital, Shanghai, China; ^2^ Department of Ophthalmology, Fudan University Eye Ear Nose and Throat Hospital, Shanghai, China

**Keywords:** varicella-zoster virus, acute retinal necrosis, aqueous humor, metalloproteinase-3, tissue inhibitors of metalloproteinase-1

## Abstract

**Objective:**

Acute retinal necrosis (ARN) caused by varicella-zoster virus (VZV) is associated with changes in specific proteins in the eye’s fluid, particularly matrix metalloproteinase-3 (MMP-3), an enzyme that breaks down tissue structures, and tissue inhibitor of metalloproteinase-1 (TIMP-1), which regulates MMP activity. This study aims to investigate how these proteins correlate with the progression of ARN.

**Methods:**

We analyzed aqueous humor samples from 33 patients with ARN and 23 control patients with virus-negative uveitis. MMP-3 levels were measured using immunoturbidimetry, and TIMP-1 levels were determined using an enzyme-linked immunosorbent assay. We examined the relationships between these protein levels and clinical findings using statistical correlation methods.

**Results:**

MMP-3, TIMP-1 were significantly higher in the aqueous humor of ARN patients compared to the controls (P<0.0001). Correlation analysis revealed a significant correlation between MMP-3 levels and TIMP-1 (r = 0.460, P = 0.007). The upregulation of MMP-3 and TIMP-1 was found to parallel VZV DNA load and IL-6 levels. Additionally, they exhibited negative correlation with best corrected visual acuity (BCVA) and positive correlation with the percentage of active retinal necrosis area.MMP-3 was markedly enhanced in all 14 cases of retinal detachment (RD), whereas TIMP-1 levels were significantly reduced in the same cohort of eyes. Patients with initial higher TIMP-1 levels have a significantly increased risk of developing RD, with a hazard ratio (HR) of 3.152 (95% CI, 1.082-9.18).

**Conclusion:**

The imbalance between MMP-3 and TIMP-1 may play a critical role in the development and severity of ARN. Measuring these proteins in the eye’s aqueous humor could be valuable for assessing disease progression and guiding treatment strategies, potentially improving outcomes for patients with virus-induced retinal diseases.

## 1 Introduction

Varicella-zoster virus (VZV) is a common human pathogen responsible for a variety of diseases, including varicella (chickenpox) and herpes zoster (shingles). In certain cases, VZV can lead to severe ocular complications, such as acute retinal necrosis (ARN), a rare but devastating infectious disease of the retina. ARN is characterized by necrotizing retinitis, occlusive retinal vasculitis, and vitritis, which often result in significant vision loss. Despite timely diagnosis and treatment, ARN is frequently associated with a high risk of retinal detachment (RD), contributing to poor visual outcomes (Anthony et al., 2020; Laíns and Eliott, 2022). This condition can affect individuals across a wide age range, from 15 to 75 years old, with no clear gender predilection. Although typically unilateral, bilateral involvement may occur in certain cases (Meghpara et al., 2010).

Among the viruses that can trigger ARN, VZV is the most common causative agent (Ganatra et al., 2000), which is a neurotropic alphaherpesviruse that establishes latency in neural ganglia following initial infection. Reactivation of VZV typically occurs with a decline in cellular immunity, often due to aging, immunosuppressive therapy, or underlying immune compromise. The pathogenesis of ARN involves a complex interplay between viral infection and the host’s immune response, with both viral and host factors contributing to disease progression. The mechanisms underlying this interplay remain incompletely understood, whether the virus directly infects the retina cells due to immunosuppression or the immune system overreacts to the pathogens is still under debate, and further research is critical to elucidating these interactions at the molecular level. Identifying key factors that drive this process could have significant implications for the treatment and prognosis of ARN, and ultimately, for advancing human health.

Matrix metalloproteinases (MMPs), a family of metal ion-dependent endopeptidases, play a crucial role in the degradation of extracellular matrix (ECM) components, including various collagens and proteins. MMPs are typically upregulated in intraocular endothelial and immune cells in response to pathological processes like inflammation and angiogenesis (Lambert et al., 2002; Giebel et al., 2005). These enzymes are tightly regulated by tissue inhibitors of metalloproteinases (TIMPs), which bind non-covalently to the active sites of MMPs, thereby inhibiting their enzymatic activity. Together, MMPs and TIMPs are key modulators of the extracellular matrix and are believed to play important roles in retinal diseases (Andrew et al., 2021). However, there is a significant gap in the literature regarding the expression of MMPs and TIMPs in ARN.

In this study, we aimed to quantitatively assess the levels of MMP-3 and TIMP-1 in the aqueous humor (AH) of ARN patients, using virus-negative uveitis (UV) patients as a control group. Our goal was to explore the potential correlation between the expression levels of these molecules and viral infection, cytokine activity, and the clinical manifestations of ARN. By understanding these relationships, we hope to contribute to the development of novel diagnostic and therapeutic strategies, ultimately improving the management of ARN and its impact on human health.

## 2 Materials and methods

### 2.1 Diagnosis criteria and data compilation

This retrospective observational study reviewed the clinical records of 33 patients (19 men and 14 women) diagnosed with ARN, who visited our clinic between December 2017 and November 2019. All patients met the diagnostic criteria for ARN as defined by the American Uveitis Society (Holland, 1994). A qualitative multiplex PCR assay was conducted at the first visit to detect the genomic DNA of herpes simplex virus types 1 and 2, VZV, Epstein–Barr virus, and cytomegalovirus (CMV). If VZV was detected, a real-time qPCR assay was subsequently performed. All patients were followed for up to 12 months from the initiation of treatment, with follow-up discontinued upon the occurrence of RD. The mean age of the ARN group was 51.7 ± 10.9 years, ranging from 16 to 71 years. The control group comprised of 23 eyes from 23 virus-negative uveitis patients, including 13 males and 10 females, aged between 14 and 70 years, with a mean age of 45.7 ± 15.6 years, none of whom experienced RD. There were no statistically significant differences in age or gender distribution between the ARN and control groups (P > 0.05), indicating comparability between the two groups. This study adhered to the principles of the Declaration of Helsinki and received approval from the Institutional Review Board of the Eye and ENT Hospital of Fudan University.

Best corrected visual acuity (BCVA) and ultra-widefield (UWF) fundus imaging were performed on the affected eyes. UWF imaging was carried out using the Optos 200Tx laser scanning ophthalmoscope retinal imaging system (Optos, UK). Two experienced physicians independently assessed the UWF images, reaching a consensus on the percentage of active retinal necrosis area. Additionally, parameters such as disease duration and intraocular pressure (IOP) were recorded.

### 2.2 Sample collection and analysis

Upon diagnosed, all patients received timely and standardized antiviral therapy. AH samples (100 μl) were collected before the first injections of ganciclovir. These samples were frozen and stored at -80°C, along with control group samples. MMP-3 levels in the aqueous humor were measured using latex immunoturbidimetry with commercially available kits from Precise Biotechnology Co., Ltd. (Suzhou, China). The detection was performed with an automatic biochemical analyzer (Cobas 702, Roche Diagnostics GmbH, Germany). TIMP-1 concentrations were measured according to the manufacturer’s instructions using an Enzyme-Linked Immunosorbent Assay (ELISA, R&D Systems, Minneapolis, Minnesota, USA). The real-time qPCR assay was conducted using a TaqMan PCR kit (Shanghai ZJ BioTech Co., Ltd., Shanghai, China) on a real-time PCR system (ABI 7500, Thermo Fisher Scientific, Waltham, MA). Viral DNA load was expressed as the number of copies per milliliter of aqueous humor, with a detection threshold of 1 × 10^3^ copies/mL. IL-6 levels in the aqueous humor were determined by Electro-Chemiluminescence (ECL) immunoassay (Cobas e601, Roche Diagnostics GmbH, Germany).

### 2.3 Protein structure analyses

Protein structure of MMP3/TIMP-1 complex (Gomis-Rüth et al., 1997) (1uea) is from Protein Data Bank (PDB). ChimeraX from UCSF (Meng et al., 2023) was used to analyze and visualize the interface between MMP3 and TIMP-1. The hydrogen bond interactions take advantage of the analytical capabilities built into ChimeraX.

### 2.4 Statistical analyses

Continuous data were presented as means ± standard deviations, while non-normally distributed data were expressed as median (interquartile range). Independent sample t-tests were used for comparisons between groups. Spearman’s correlation coefficient was used for correlation analysis, with *r* indicating the strength of the correlation: |*r*|≥0.8 signifies a high correlation, 0.5-0.8 a moderate correlation, 0.3-0.5 a low correlation, and <0.3 no correlation. Time-to-event data were analyzed using the Kaplan-Meier method and log-rank test. A *P*-value of < 0.05 was considered statistically significant.

## 3 Results

### 3.1 Clinical characteristics of subjects

The study included a total of 33 ARN patients, comprising 19 males (58%) and 14 females (42%), with a mean age of 51.7 ± 10.9 years (range: 16–71 years). The duration between symptom onset and presentation to the clinic was 17.2 ± 6.2 days, ranging from 7 to 30 days. The baseline best corrected visual acuity (BCVA) was 0.20 ± 0.24 (range: 0.00–1.00), while intraocular pressure (IOP) was measured at 14.2 ± 5.0 mmHg (range: 7.1–27.9 mmHg). The percentage of active retinal necrosis area averaged 37.92 ± 22.92%, with a range from 1.47% to 81.20%. The viral load of VZV DNA in the aqueous humor was 7.8 × 10^7^ ± 1.2 × 10^8^ copies/mL (range: 1.1 × 10^5^ to 6.0 × 10^8^ copies/mL). Additionally, the levels of IL-6 in the aqueous humor were 7614 ± 10535 pg/mL, with a range from 111 to 55489 pg/mL. No primary or secondary immune deficiencies, such as Severe Combined Immunodeficiency (SCID), Wiskott-Aldrich Syndrome (WAS), cancer, organ transplantation, or HIV infection/AIDS, were found in any of the patients. Detailed demographic and baseline clinical data of ARN patients are presented in **Table 1**.

**Table 1:** Baseline characteristics of the ARN patients.

Gender	19 males (58%); 14 females (42%)
Age(years)	51.7 ± 10.9 (range, 16–71)
Duration between symptoms and presentation(days)	17.2 ± 6.2 (range, 7–30)
BCVA	0.20 ± 0.24(range, 0.00–1.00)
IOP (mmHg)	14.2 ± 5.0 (range, 7.1–27.9)
Percentage of active retinal necrosis area (%)	37.92 ± 22.92 (range, 1.47–81.20)
VZV DNA load (copies/mL)	7. 8 × 10^7^ ± 1. 2 × 10^8^ (range, 1.1 × 10^5^–6. 0 × 10^8^)
IL-6 (pg/mL)	7614 ± 10535 (range, 111–55489)

### 3.2 Protein analysis of MMP-3/TIMP-1 complex

**Figure 1:**
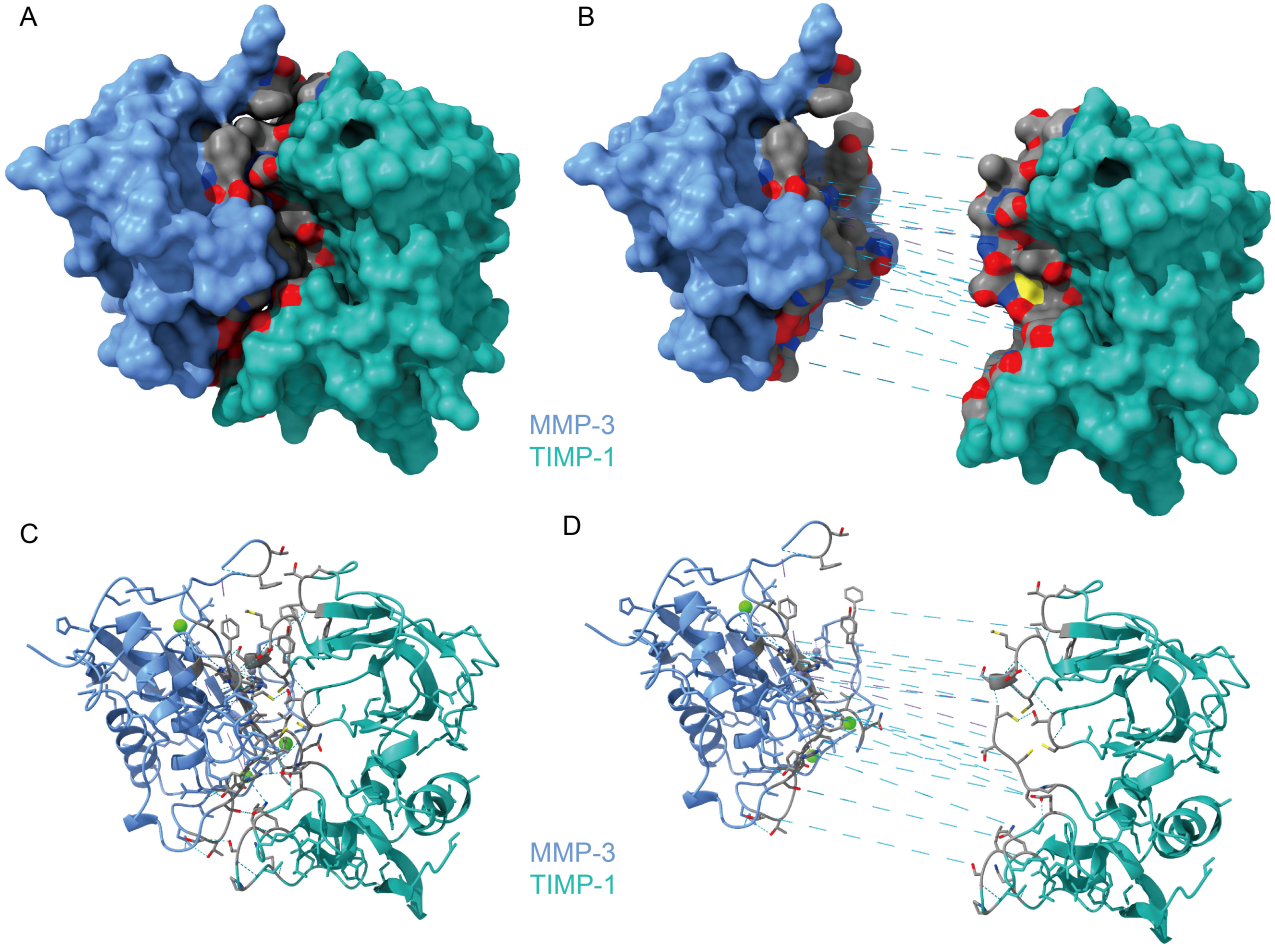
The Protein Structure of MMP-3/TIMP-1 Complex. **(A)** The overview of MMP-3/TIMP-1 Complex. **(B)** The interface between MMP-3 and TIMP-1 by exploding MMP3 and TIMP-1. The gray area is the interaction surface. And the dashed blue line is hydrogen bonds. **(C)** Cartoon presentation of the whole complex of MMP-3/TIMP-1. **(D)** Cartoon presentation of the interaction surface.

We downloaded the structure of the complex from the PDB (1uea) and re-analyzed the interaction between MMP-3 and TIMP-1 (**Figure 1**). The interaction between MMP-3 and TIMP-1 is primarily mediated through the N-terminal segment of TIMP-1. The Cys 1 (cysteine) residue in TIMP-1 coordinates with the catalytic zinc ion in MMP-3, effectively blocking the active site. The Thr 2 (threonine) residue of TIMP-1 extends into the specificity pocket of MMP-3, where its β-methyl group fits into a hydrophobic niche, while the β-hydroxy group interacts with the polar environment at the pocket’s entrance (Gomis-Rüth et al., 1997). These interactions, along with other hydrophobic contacts, stabilize the TIMP-1/MMP-3 complex and inhibit the proteolytic activity of MMP-3. Therefore, it is reasonable to speculate that when the balance of these two proteins in aqueous humor is disrupted, this inhibition is also affected.

### 3.3 MMP-3 levels correlated with TIMP-1 in the aqueous humor

**Figure 2:**
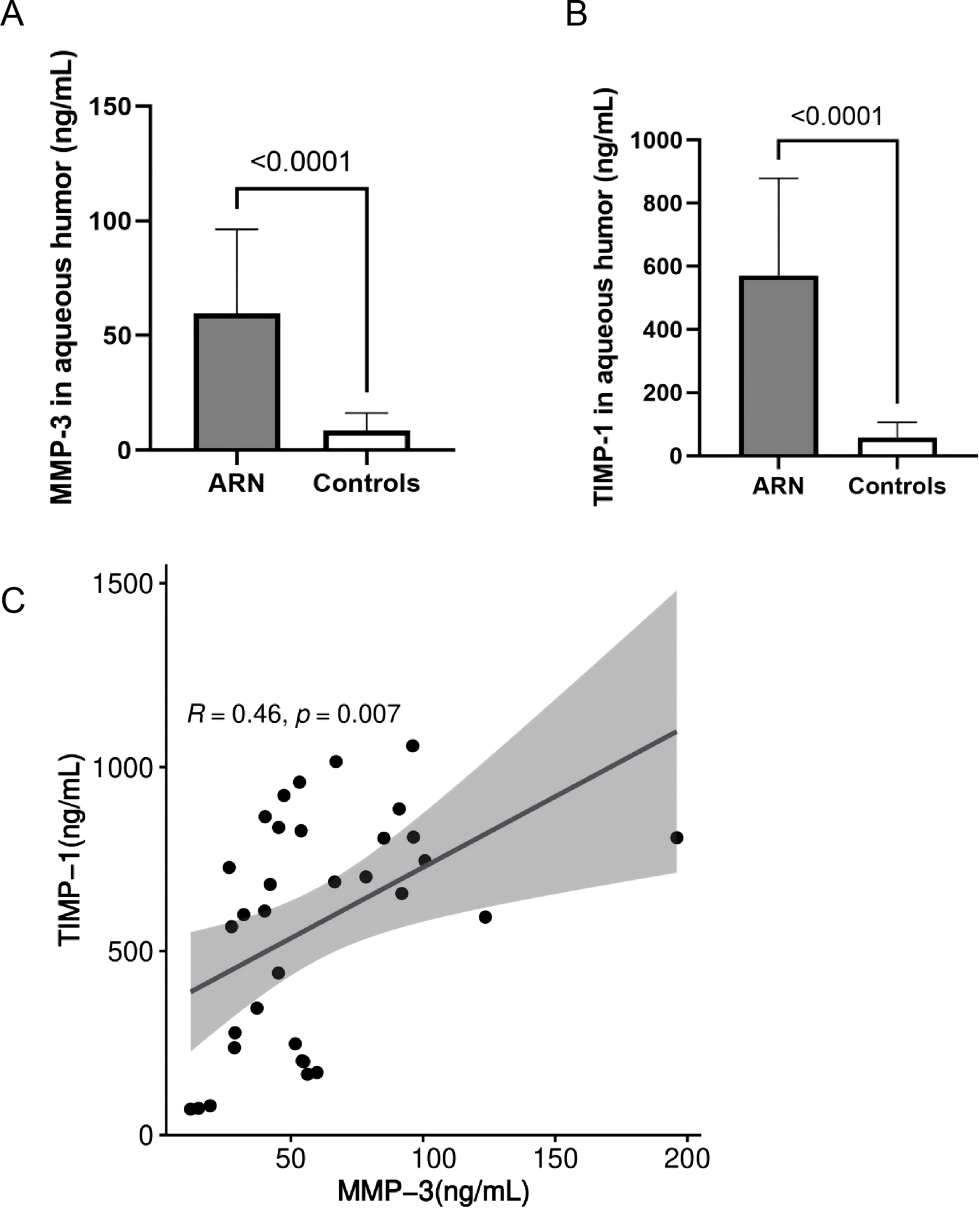
Comparison and Correlation of MMP-3 and TIMP-1 Levels in the AH Between Control and ARN Patients. MMP-3 **(A)**, TIMP-1 **(B)** were significantly higher in the AH of ARN patients compared to the controls. (*P*<0.0001). **(C)** Correlation analysis between MMP-3 and TIMP-1 were performed using linear regression.

The concentration of MMP-3 in the aqueous humor of ARN patients prior to treatment was 59.6 ± 36.7 ng/mL, with a median value of 53.3 ng/mL (**Figure 2A**). In contrast, the control group exhibited significantly lower MMP-3 levels, averaging 8.6 ± 7.6 ng/mL (*P* < 0.0001). This marked difference underscores the significant upregulation of MMP-3 in the context of ARN, likely reflecting the heightened inflammatory and pathological processes associated with the disease.

Similarly, the levels of TIMP-1 in the AH of ARN patients were also significantly elevated. The mean concentration of TIMP-1 in the ARN group was 571.6 ± 307.1 ng/mL, with a median value of 656.3 ng/mL (**Figure 2B**). In contrast, the control group had considerably lower TIMP-1 levels, averaging 58.8 ± 47.2 ng/mL. The substantial increase in TIMP-1 levels in ARN patients suggests a robust response mechanism, potentially linked to the regulatory role of TIMP-1 in maintaining extracellular matrix (ECM) stability and controlling MMP activity under disease conditions.

**Figure 2** visually compares the concentrations of MMP-3 and TIMP-1 between the ARN and control groups, highlighting the significant disparities observed. Further statistical analysis using linear regression revealed a significant positive correlation between MMP-3 and TIMP-1 levels (*r* = 0.460, *P* = 0.007, **Figure 2C**). This correlation suggests that in the disease state, both MMP-3 and TIMP-1 are upregulated in tandem, possibly reflecting a coordinated response to viral infection and inflammation in ARN. The linear relationship between these two proteins reinforces the notion that the dysregulation of MMPs and TIMPs may play a crucial role in the pathophysiology of ARN, contributing to the progressive nature of the disease and the associated tissue damage.

### 3.4 Relationship between aqueous humor outcome and ophthalmic parameters in ARN patients

Spearman correlation analysis was conducted to evaluate the relationship between AH outcome and various ophthalmic parameters in ARN patients. Correlation analysis revealed that MMP-3 correlated significantly with TIMP-1, VZV DNA load and IL-6. The analysis revealed statistically significant negative correlation between MMP-3, TIMP-1 levels and best corrected visual acuity (BCVA) (*P* = 0.0154, 0.0043), suggesting that higher MMP-3, TIMP-1 levels are associated with decreased visual function. Additionally, positive correlation was observed between MMP-3, TIMP-1 levels and the percentage of active retinal necrosis area (*P* = 0.0455, 0.0049), indicating that elevated MMP-3, TIMP-1 may reflect more extensive retinal damage.

No significant correlations were found between MMP-3, TIMP-1 levels and intraocular pressure (IOP) or disease duration (*P* > 0.05), suggesting that MMP-3, TIMP-1 may not be directly influenced by these factors. The relationships between MMP-3, TIMP-1 levels and clinical characteristics in ARN patients are summarized in **Table 2**, highlighting the potential of MMP-3, TIMP-1 as a biomarker for disease severity and visual prognosis.

**Table 2:** Relationship between AH outcome and ophthalmic parameters.

		MMP-3		TIMP-1		VZV DNA load		IL-6	
		*r*	*P* value	*r*	*P* value	*r*	*P* value	*r*	*P* value
AH outcome	MMP-3	–	–	0.478	0.0049	0.403	0.0199	0.595	0.0003
	TIMP-1	0.478	0.0049	–	–	0.842	<0.0001	0.763	<0.0001
	VZV DNA load	0.403	0.0199	0.842	<0.0001	–	–	0.745	<0.0001
	IL-6	0.595	0.0003	0.763	<0.0001	0.745	<0.0001	–	–
Ophthalmic parameters	BCVA	-0.453	0.0154	-0.522	0.0043	-0.43	0.0223	-0.296	0.1332
	IOP (mmHg)	0.0395	0.8420	-0.0449	0.8204	0.0107	0.9569	0.0315	0.8762
	Disease duration (days)	0.204	0.2807	-0.0613	0.7477	0.105	0.5803	0.0371	0.8486
	Retinal zones involved (%)	0.374	0.0455	0.508	0.0049	0.517	0.0041	0.461	0.0135

### 3.5 Molecular alterations associated with RD in ARN patients

Retinal detachment (RD) occurred in 22 out of 33 eyes (67.0%) affected by ARN, and all were subsequently treated with vitrectomy. Aqueous humor samples were collected from 14 of these patients prior to the operation. Analysis of the viral DNA load using a paired t-test revealed a significant decrease in VZV DNA levels across all 14 cases (*P* = 0.0002), indicating a reduction in viral presence following treatment.

Interestingly, MMP-3 levels in the aqueous humor were markedly increased in all 14 eyes (100% increase), suggesting an enhanced enzymatic activity that may be related to ongoing inflammatory processes or tissue remodeling in response to the RD. Conversely, the levels of tissue inhibitor of metalloproteinase-1 (TIMP-1) were significantly reduced in the same cohort of eyes (100% decrease), pointing to a disruption in the balance between MMPs and their inhibitors. This imbalance was further highlighted by the elevated MMP-3/TIMP-1 ratio observed in all 14 eyes (100% increase), which could indicate a shift toward a more degradative environment within the ocular tissues, potentially contributing to the pathophysiology of RD.

**Figure 3:**
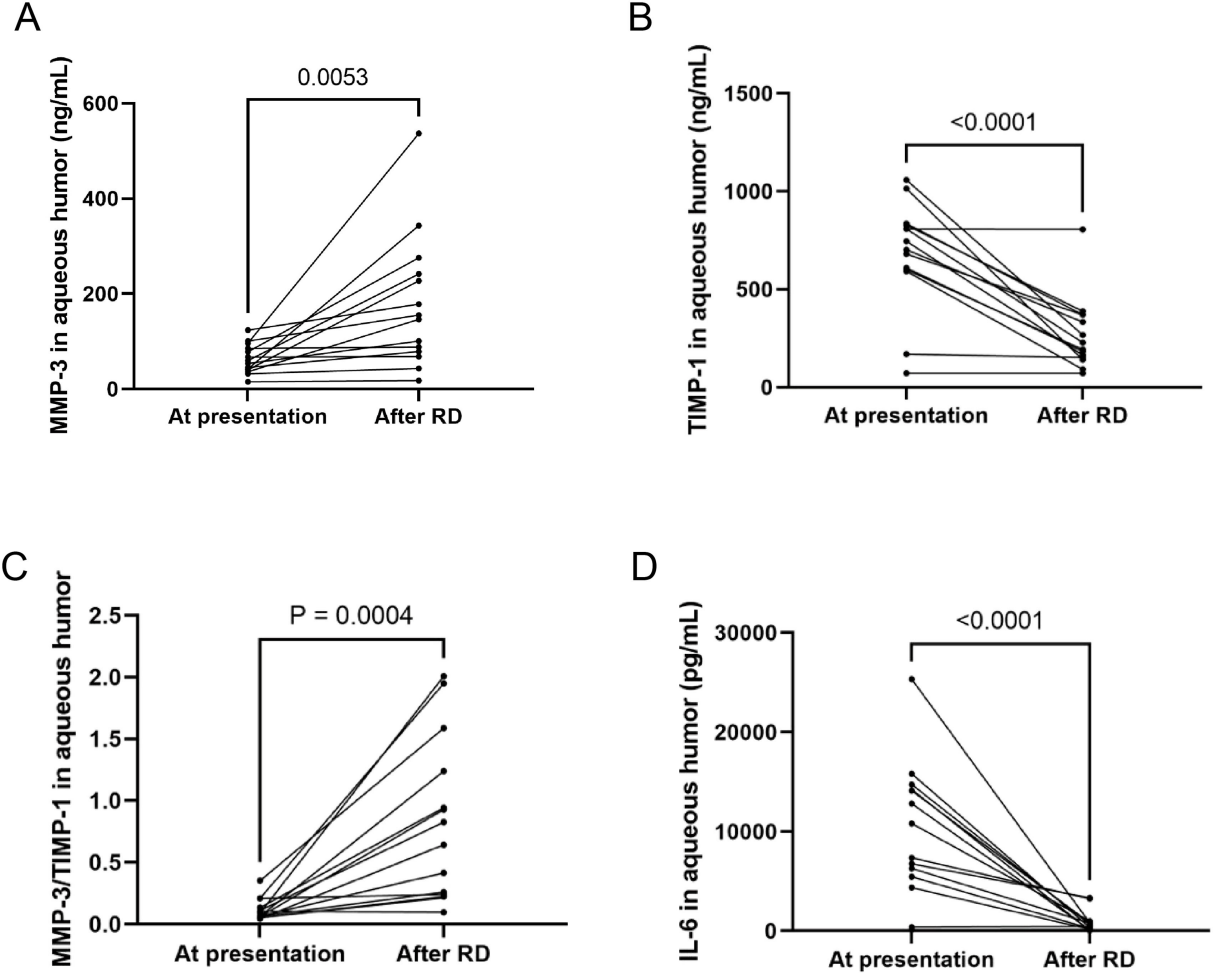
MMP-3, TIMP-1, MMP-3/TIMP-1, IL-6 at presentation and after RD. **(A)** MMP-3 levels in the AH were increased in all 14 eyes. **(B)** TIMP-1 were significantly reduced in all eyes. **(C)** MMP-3/TIMP-1 ratio was elevated in all eyes. **(D)** IL-6 levels in AH decreased in all patients except one eye.

Additionally, IL-6 levels in the aqueous humor showed a decrease in all patients except for a slight increase in one eye, reflecting a general reduction in inflammatory cytokine levels post-treatment. The changes in MMP-3, TIMP-1, the MMP-3/TIMP-1 ratio, and IL-6 levels before and after the occurrence of RD are illustrated in **Figure 3**. These findings provide insights into the molecular alterations associated with RD in ARN patients and underscore the potential role of MMP-3 and TIMP-1 in the progression and management of this condition.

### 3.6 Correlation between TIMP-1 levels and the risk of RD

**Figure 4:**
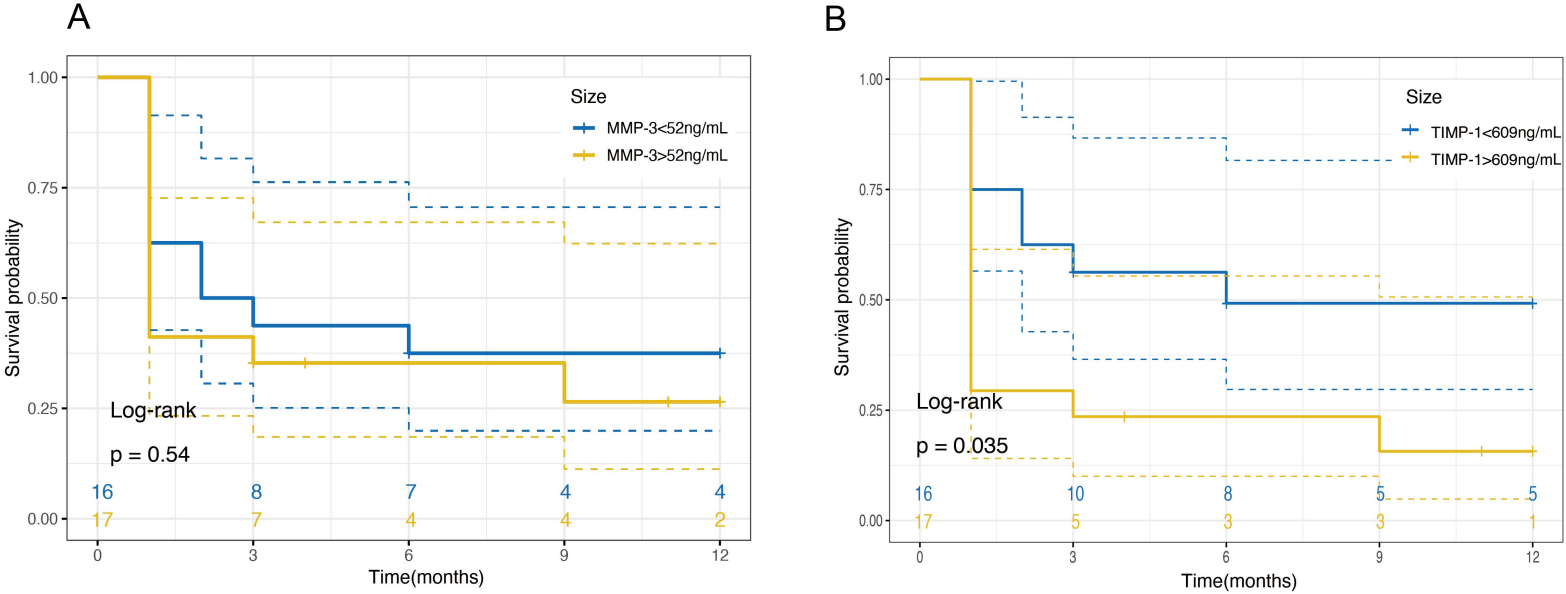
Kaplan-Meier survival curves for retinal detachment free survival based on MMP-3 and TIMP-1 Levels in Aqueous Humor. **(A)** Survival probability over 12 months for patients categorized by MMP-3 levels in aqueous humor. Patients are divided into two groups based on MMP-3 concentrations: ≤52ng/mL (blue line) and >52ng/mL (yellow line). The plot shows no significant difference in the survival probabilities between the two groups over the time period, as indicated by a log-rank test *P*-value of 0.54. **(B)** Survival probability over 12 months for patients categorized by TIMP-1 levels in aqueous humor. Patients are divided into two groups based on TIMP-1 concentrations: ≤609ng/mL (blue line) and >609ng/mL (yellow line). The plot indicates a significant difference in survival probabilities, with higher TIMP-1 levels associated with a decreased survival free of retinal detachment, as evidenced by a log-rank test *P*-value of 0.035.

In terms of RD-free survival, the Kaplan–Meier curve demonstrated a significant correlation between the initial aqueous humor TIMP-1 levels and the risk of retinal detachment (RD) (**Figure 4B**; *P* = 0.035). Patients with higher TIMP-1 levels were found to have a significantly increased risk of developing RD, with a hazard ratio (HR) of 3.152 (95% CI, 1.082-9.18). This suggests that elevated TIMP-1 levels may serve as a predictive marker for RD in ARN patients. In contrast, MMP-3 levels did not show a significant association with RD risk (**Figure 4A**; *P* = 0.54).

## 4 Discussion

To our knowledge, this is the first study to investigate MMP-3 and TIMP-1 levels in the AH of patients with VZV-induced ARN, tracking changes from initial to final sampling. Our findings demonstrate that VZV-induced ARN is associated with significantly elevated levels of MMP-3 and TIMP-1 in the AH. Further correlation analysis revealed a significant association between these elevated levels and the severity of the disease.

MMPs play a crucial role in various ophthalmic diseases, primarily by regulating both physiological and pathological processes in the eye. Previous studies have shown that MMPs are either absent or expressed at low levels in normal tissue (Pepper, 2001), but become upregulated in endothelial and immune cells in response to inflammation and angiogenesis (Lambert et al., 2003). Recent research has indicated that MMP-3 is overexpressed in various virus-induced diseases (Lind et al., 2019; Filiberti et al., 2021). Our quantitative analysis, conducted on a large sample size of VZV-induced ARN patients, revealed significantly elevated levels of MMP-3 and TIMP-1 in the aqueous humor compared to virus-negative uveitis cases presenting with retinal inflammation. The findings suggest a potential association between MMP-3/TIMP-1 dynamics and different immune and inflammatory pathologies in VZV-induced ARN. It has been reported that MMP-3 exhibits broad-spectrum antiviral activities, enhancing host antiviral immunity to restrict viral infections (Feng et al., 2022). The increase in MMP-3 levels in aqueous humor of ARN patients may therefore be related to this antiviral mechanism. TIMP-1, the natural inhibitor of MMP-3, plays a significant role in controlling cellular behavior, tissue remodeling, and ECM turnover. The exogenous application of TIMP-1 has been reported to significantly modulate the mosaics and restore their homogeneity in the retina of the rat model of retinitis pigmentosa and may be a potential therapeutic agent (Ji et al., 2014). The loss of balance between MMP and TIMP activity is a key feature of the pathophysiology of several diseases (Mukherjee and Das, 2024). Our findings demonstrate a significant linear correlation between MMP-3 and TIMP-1 during the early stage of ARN, which may be related to the mechanism that maintains the MMP/TIMP balance in the eye.

Our findings suggest that measuring TIMP-1 levels in the aqueous humor could have significant clinical implications. Elevated TIMP-1 levels were associated with a higher risk of RD, indicating that TIMP-1 could serve as a prognostic biomarker for disease progression in ARN patients. Incorporating TIMP-1 measurement into routine clinical practice may aid in early identification of patients at increased risk for RD, allowing for timely interventions and tailored treatment strategies. This could include closer monitoring, earlier surgical consideration, or the exploration of therapies aimed at modulating TIMP-1 activity to restore the balance between MMPs and TIMPs.

Prior to this study, little was known about the MMPs or TIMPs responses during VZV infection in ARN. MMP-3 has been reported as an intercellular signaling molecule that modulates neuroinflammatory responses (Kim et al., 2005). VZV-induced ARN often presents initially as central retinal artery occlusion and posterior ischemic optic neuropathy (Allan et al., 2023). Both stressed and apoptotic neurons release MMP-3, which triggers microglial activation and the production of inflammatory cytokines (Candelario-Jalil et al., 2009). Microglia are considered the primary source of MMPs in retinal tissues (Chen and Xu, 2015). Furthermore, due to its transcription factor-like action, MMP-3 can increase the synthesis of other MMPs. This cascade effect may reflect a heightened neuroinflammatory response in ARN patients. Previous reports have indicated that the initial retinal zones affected by VZV correlate with viral load in ARN, which aligns with our findings from a larger sample size (Lei et al., 2021). The larger the zone of retinal necrosis, the more severe the neuronal injury and inflammatory response in ARN patients, leading to increased infiltration of inflammatory cells and expression of inflammatory mediators. A recent study has also found that ECM remodeling alters mitochondrial homeostasis in an evolutionally conserved manner, suggesting that ECM-mitochondrial crosstalk is an ancient immune pathway capable of detecting ECM damage caused by infection or mechanical stress (Zhang et al., 2024). ECM remodeling induced by increased intraocular MMP-3 may trigger a range of immune and metabolic responses.

Interestingly, our study observed that following antiviral treatment and a significant reduction in VZV DNA load, MMP-3 levels continued to increase while TIMP-1 levels decreased during RD progression. This suggests that the imbalance between MMP-3 and TIMP-1 is not solely dependent on active viral replication but may be driven by ongoing pathogenic mechanisms such as persistent inflammation and ECM remodeling. The elevated MMP-3 could contribute to degradation of retinal structural components, weakening the retinal architecture and facilitating RD. Concurrently, decreased TIMP-1 levels may fail to counteract the proteolytic activity of MMP-3, exacerbating tissue damage. These findings imply that therapeutic strategies targeting the MMP-3/TIMP-1 axis might be necessary even after viral clearance to prevent RD.

The vitreous humor primarily consists of collagen and glycosaminoglycans, while the vascular wall of the retina is mainly composed of type IV collagen (Azuma et al., 1998). MMPs are unique proteinases capable of hydrolyzing fibrous collagen, enabling extensive degradation of the ECM and the intact type IV collagen basement membranes. Activated MMP-3 can interact with ECM proteins such as fibronectin (FN) and laminin (LN) in the retina (Ocklind, 1998), subsequently activating other MMPs that collectively contribute to ECM degradation. Previous studies have observed elevated levels of stromelysin-1 (MMP-3) in the vitreous and subretinal fluid during rhegmatogenous retinal detachment (RRD) (Symeonidis et al., 2007, 2011), findings consistent with our observations in the aqueous humor of ARN patients. After antiviral therapy, a significant proportion of ARN patients still experience RD, accompanied by decreased viral load and interleukin-6 levels in the AH. Conversely, MMP-3 expression increases while TIMP-1 levels decrease. In our investigation, TIMP-1 levels in the aqueous humor emerged as a predictor for the risk of RD, suggesting that the disruption of MMPs-TIMP-1 homeostasis may play a crucial role in creating a permissive environment for RD. These findings offer new insights into the potential mechanisms underlying RD and may be valuable in developing protective measures for patients with ARN.

It’s important to acknowledge several limitations in this study. Firstly, we had insufficient cases of ARN caused by other virus types (such as HSV and CMV) to perform a comparative analysis of MMP-3 and TIMP-1 expression across different viral strains. Secondly, only MMP-3 and TIMP-1 were detected, leaving the involvement of other MMPs and TIMPs in ARN and the pathological mechanisms underlying ARN-induced RD unknown. Additionally, since the control group does not include cases of retinal necrosis unrelated to viral infections or non-viral RD, we cannot confirm whether the changes in MMP-3 and TIMP-1 we observed should be directly attributed to viral infection or are common features of retinal necrosis of various etiologies and non-viral RD. To address these limitations, we plan to expand our study to include more distinct etiological subgroups and a broader range of biomarkers, allowing for a deeper investigation into the role of MMPs-TIMPs mechanisms in ARN pathogenesis.

## 5 Conclusion

Our findings indicate that the levels of MMP-3 and TIMP-1 in the AH may serve as valuable markers for assessing the severity and prognosis of ARN, offering new insights into the pathology of the disease. Measuring TIMP-1 levels could have significant clinical implications, aiding in early identification of patients at increased risk for RD and informing timely interventions. Furthermore, understanding the imbalance between MMP-3 and TIMP-1, and its relationship with inflammatory mediators like IL-6, opens potential avenues for therapeutic strategies targeting the MMP-3/TIMP-1 axis. Further investigation into the role of MMPs and TIMP-1 in the disease processes of ARN could provide novel perspectives and potential avenues for disease treatment and intervention.

## Data Availability

The original contributions presented in the study are included in the article/supplementary material. Further inquiries can be directed to the corresponding author.
